# Classification of Pancreatic Cancer and Normal Tissue in 2D and 3D Optical Coherence Tomography Images Using Convolutional Neural Networks: A Comparative Study

**DOI:** 10.3390/cancers18050732

**Published:** 2026-02-25

**Authors:** Maria Druzenko, Bastian Westerheide, Caroline Girmen, Niels König, Robert Schmitt, Svetlana Warkentin, Katharina Jöchle, Sebastian Cammann, Georg Wiltberger, Martin W. von Websky, Thomas Vogel, Florian W. R. Vondran, Iakovos Amygdalos

**Affiliations:** 1 Department of General, Visceral, Pediatric and Transplantation Surgery, University Hospital RWTH Aachen, Pauwelsstrasse 30, 52074 Aachen, Germany; 2 Fraunhofer Institute for Production Technology IPT, Steinbachstraße 17, 52074 Aachen, Germany; 3 Laboratory for Machine Tools and Production Engineering (WZL) of RWTH Aachen University, Campus-Boulevard 30, 52074 Aachen, Germany; 4 Institute for Pathology, University Hospital RWTH Aachen, Pauwelsstrasse 30, 52074 Aachen, Germany

**Keywords:** optical coherence tomography, pancreatic ductal adenocarcinoma, artificial intelligence, convolutional neural networks

## Abstract

Background/Objectives: Early and complete (R0) surgical resection is essential for optimal outcomes in pancreatic cancer. Optical coherence tomography (OCT) combined with artificial intelligence (AI) may offer real-time intraoperative guidance, potentially reducing reliance on frozen sections. This ex vivo study evaluated convolutional neural networks (CNNs) for distinguishing pancreatic ductal adenocarcinoma (PDAC) from normal pancreatic tissue in OCT images obtained ex vivo. Methods: Between October 2020 and April 2021, OCT scans were obtained from resected pancreatic specimens of 27 adult patients. Tumor and adjacent normal tissue were imaged using a 1310 nm OCT system, followed by histopathological confirmation. A total of 25 PDAC and 30 non-malignant scans were preprocessed and analyzed using cross-validated CNN models (ResNet50, DenseNet121, and MobileNetV2) with both 2D and 3D inputs. Results: Using five-fold stratified cross-validation on 9040 2D and 3000 3D samples (224 px resolution), the 3D DenseNet121 model achieved the highest performance, with an F1-score of 0.74, sensitivity of 72%, and specificity of 81%. Other architectures demonstrated comparable results. Conclusions: AI-assisted OCT can accurately differentiate PDAC from normal pancreatic tissue ex vivo, supporting its potential as a rapid intraoperative diagnostic adjunct. Further studies are warranted to assess its in vivo performance and utility in evaluating resection margins.

## Simple Summary

Surgeons treating pancreatic cancer need to remove all cancer tissue to give patients the best chance of recovery. This study looked at whether a special imaging method, called optical coherence tomography (OCT), combined with artificial intelligence (AI), could tell cancer tissue apart from normal pancreatic tissue, which could be used to check if the entire tumor has been removed during surgery. Researchers scanned tissue that had already been removed from 27 patients with pancreatic cancer. They then trained computer programs to recognize differences between cancer and healthy tissue in these images. The best-performing program correctly identified cancer tissue most of the time and was also good at recognizing normal tissue. The results suggest that combining OCT with AI could one day help surgeons quickly check tissue during operations, possibly reducing the need for time-consuming laboratory tests. More research is needed to see how well this works during real surgeries on living patients.

## 1. Introduction

Pancreatic ductal adenocarcinoma (PDAC) is the 14th most common malignant neoplasm and the 7th leading cause of cancer-related death worldwide [1]. Its high mortality primarily stems from late diagnosis and the lack of effective treatment options for advanced disease. In Western Europe, the 5-year survival rate remains dismal, ranging between 0.5% and 9% [2]. Early detection (T1, N0, M0) is crucial for curative treatment but is rare due to nonspecific symptoms and the absence of reliable screening methods. Approximately 60–70% of tumors arise in the pancreatic head [3], where proximity to major vessels and bile ducts often leads to local invasion, rendering many tumors unresectable.

Complete (R0) surgical resection offers the best oncologic outcomes in pancreatic cancer [4]. Depending on tumor location and extent, surgical approaches include left pancreatic resection for tumors in the body or tail, the classical pancreatoduodenectomy (Kausch–Whipple procedure), or the pylorus-preserving Traverso–Longmire method for lesions of the pancreatic head [4]. Achieving an R0 resection requires accurate intraoperative margin assessment, currently performed by frozen section analysis. Although reliable, this technique is time-consuming and costly, extending operative duration and potentially increasing postoperative complications and hospital stay [5]. Given reported non-R0 resection rates ranging from <20% to >80% [6], more efficient and precise intraoperative margin assessment methods are urgently needed.

Optical coherence tomography (OCT) provides high-resolution, real-time imaging of tissue microstructures and represents a promising alternative [7,8,9,10]. When integrated with artificial intelligence (AI), particularly convolutional neural networks (CNN), OCT may enable automated and accurate tissue classification [11,12]. Previous studies have demonstrated the diagnostic potential of OCT combined with CNN—for instance, our group successfully applied this approach to differentiate colorectal liver metastases and cholangiocarcinoma from normal liver tissue [8,9,11]. However, existing studies on OCT for pancreatic tissue have largely excluded AI-based analysis [13,14,15,16,17,18].

This study investigates the feasibility of combining two- (2D) and three-dimensional (3D) OCT with CNN to differentiate PDAC from healthy pancreatic tissue ex vivo. We evaluate three established CNN architectures—ResNet50, DenseNet121, and MobileNetV2—selected for their proven performance in medical image classification, varying network complexity, and computational efficiency [19,20,21]. While 2D CNN models represent the established standard in OCT-based image analysis and have demonstrated robust performance across various clinical applications, OCT inherently provides volumetric information. PDAC is characterized by complex three-dimensional glandular and stromal architecture. We therefore hypothesized that 3D CNN may better capture spatial continuity, glandular structures, and microarchitectural distortions across adjacent slices, potentially improving tissue discrimination compared to single-slice 2D representations.

## 2. Materials and Methods

### 2.1. Patient Cohort and Inclusion Criteria

Consecutive adult patients undergoing elective pancreatic resection at University Hospital RWTH Aachen between October 2020 and April 2021 were included in this prospective study. Exclusion criteria comprised patients under 18 years, emergency procedures, and inability or unwillingness to provide informed consent.

### 2.2. Specimen Collection and Scanning

**Figure 1:**
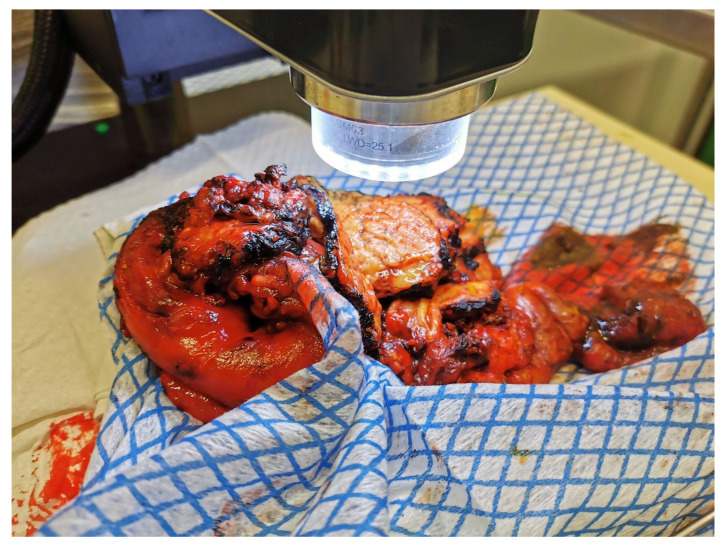
A typical OCT scanning orientation of a pancreas specimen with PDAC.

Freshly resected tissue specimens were immediately transported from the operating room to the pathology department for optimal preservation. A pathologist performed macroscopic evaluation and marked resection margins with colored ink. Following standard frozen section analysis [22], the specimens were cut into 0.5 cm slices, and OCT imaging was conducted (Figure 1). Both macroscopically healthy and suspicious regions were scanned using a table-top OCT system (Telesto™ V1, Thorlabs GmbH, Lübeck, Germany). Scanned regions were needle-marked and color-coded to ensure spatial correlation. After imaging, samples were formalin-fixed, paraffin-embedded, and stained with hematoxylin and eosin (H&E). A pathologist reviewed the histological slides to establish direct correspondence between OCT regions and histological diagnoses.

### 2.3. OCT Imaging System and Scan Settings

A spectral-domain OCT system with a central wavelength of 1310 nm was used, as described previously [7,8,9]. The system provides an axial resolution of 4.9 µm and a penetration depth of up to 2.5 mm. Each 3D volume (C-scan) covered 9.9 mm × 2.55 mm × 2.55 mm and consisted of 512 B-scans, each derived from multiple axial A-scans. The resulting voxel resolution was 2048 × 512 × 512 pixels, corresponding to 4.83 µm × 4.98 µm × 4.97 µm in the x-, y-, and z-directions, respectively. Due to hardware configuration differences during the acquisition period, lateral resolution varied between 2.5 µm, 3 µm and 4.9 µm.

To ensure comparability across scans, all volumes were resampled using 2D or 3D interpolation to a standardized spatial resolution, using the fixed axial resolution as reference. A C-scan refers to a complete three-dimensional OCT acquisition obtained from a defined tissue region. In this study, the terms scan, C-scan, and volume are used synonymously to describe a full 3D OCT dataset. Smaller image subsets were extracted from each C-scan for model training. A patch refers to a localized 2D or 3D subregion cropped from a C-scan. The term sample denotes an individual 2D or 3D patch used as input to the CNN.

### 2.4. Sample Generation

A total of 27 patients were included, yielding 55 C-scans: 30 from non-malignant pancreatic tissue and 25 from PDAC. To establish an independent test set, 18% of scans (5 non-malignant, 5 PDAC) were randomly selected and excluded from model training. The remaining 45 scans (25 healthy, 20 malignant) were used for training and cross-validation. Cross-validation and independent test splitting were performed at the scan level. Because multiple C-scans were obtained from individual patients, strict patient-level independence across splits cannot be fully guaranteed.

Due to the large size of OCT volumes, direct training on full scans was not feasible. Instead, smaller 2D and 3D patches were extracted while preserving key structural information. Regions with high contrast-to-noise ratio (CNR) were identified and selected for inclusion. An axial size of 320 pixels was chosen as optimal for information density. Extraction was performed such that a 3-pixel margin remained above the tissue surface to prevent loss of structural information. At the scan level, class distribution was balanced (30 non-malignant, 25 PDAC). Patch extraction was performed automatically using identical criteria across all scans without class-specific stratification. Therefore, patch-level distribution reflects the intrinsic tissue composition of the respective scans and was not manually adjusted.

This process yielded 11,148 2D samples (9040 training; 2108 test) and 3700 3D samples (3000 for training; 700 for testing). Preprocessing was performed individually for each C-scan without access to class labels. To mitigate inter-scan intensity variation and suppress outliers, every tenth B-scan within each C-scan was analyzed to derive scan-specific intensity statistic. For each selected B-scan, the mean of maximum intensity values per A-scan and the mean intensity values per A-scan were computed. These values were used to define lower and upper cutoff thresholds. Intensities outside these thresholds were clipped accordingly. Following intensity clipping, samples were linearly normalized to the 0–1 range. All preprocessing steps were derived solely from intra-scan intensity distributions and applies identically across training, validation, and test sets.

Non-representative samples (e.g., <50% tissue content or excessive adipose tissue) were excluded independent of diagnosis. For validation of tissue localization, grayscale images were binarized using a dynamic threshold at the 75th percentile of pixel intensities. This internal step ensured accurate tissue surface identification without altering the data provided to the CNN. Data augmentation was applied uniformly across all training samples to enhance variability and prevent overfitting. Horizontal flipping was applied with a probability of 50%. In addition, 3D samples were used to vary image contrast, with γ defined as γ = exp(β), where β was sampled from a uniform distribution U (−3, 3). After the first training epoch, samples were randomly shifted laterally to further increase dataset diversity. Shifts were sampled from a uniform distribution U (−25, 25) pixels. For 2D samples, shifts were applied along the lateral axis only.

### 2.5. Neural Network Analysis

Three convolutional neural network (CNN) architectures were evaluated: ResNet50, DenseNet121, and MobileNetV2. ResNet50 employs skip connections to preserve image features across deeper layers, serving as a standard reference model in medical imaging [20,21]. DenseNet121 connects each layer to all preceding layers, improving feature reuse and efficiency—beneficial for smaller medical datasets [20]. MobileNetV2 is optimized for computational efficiency, making it suitable for potential intraoperative applications [19]. The selected architectures represent complementary and widely established CNN design strategies in medical image analysis.

Transfer learning was implemented to enhance performance. For 2D models, ImageNet (ILSVRC) pre-trained weights were applied. For 3D models, 2D filters were extended along the third dimension to simulate pretraining effects. Model robustness was assessed using five-fold stratified cross-validation. In each iteration, four folds were used for training and one for validation, with results averaged across all folds to reduce bias and overfitting.

All models were optimized using the Adam optimizer (PyTorch v2.9.1 implementation) with default parameters and trained for 50 epochs with an initial learning rate of 1e^–4^, reduced by a factor of 0.1 after five epochs without improvement in validation loss. Early stopping was not applied. Manual reductions were also applied at 10, 15, 20, and 30 epochs based on cross-validation results. Hyperparameters were kept consistent across models to ensure comparability rather than maximize architecture-specific performance. The batch size was 32 for 2D models and 16 for 3D models (via gradient accumulation over eight mini-batches of two samples each). Weight decay was set to 0. No additional dropout layers were introduced. Models were trained on the complete training set and evaluated on the independent test set.

## 3. Results

### 3.1. Specimen Statistics

A total of 27 patients were included in the study, comprising 15 females and 12 males. Of these, 20 patients underwent pancreatoduodenectomy, yielding 41 C-scans. Five patients underwent left-sided pancreatic resection, yielding 11 C-scans, while 2 patients underwent atypical resections, producing 3 C-scans. In total, 55 C-scans were analyzed.

### 3.2. Cross-Validation and Known-Data Test Results

All CNN architectures exhibited consistent learning behavior during five-fold cross-validation. Training loss declined markedly within the first 10–15 epochs across all models. In 2D architectures, validation loss initially increased slightly before stabilizing, whereas 3D models demonstrated stable validation loss and achieved higher accuracy and F1-scores throughout training. Among the evaluated models, DenseNet achieved the best validation performance, followed by MobileNet and ResNet. In general, 3D models outperformed 2D models, displaying superior generalization and lower inter-fold variability. However, increasing the lateral sample size in 3D inputs resulted in higher loss and reduced accuracy, suggesting diminishing returns beyond the optimal patch size.

**Figure 2:**
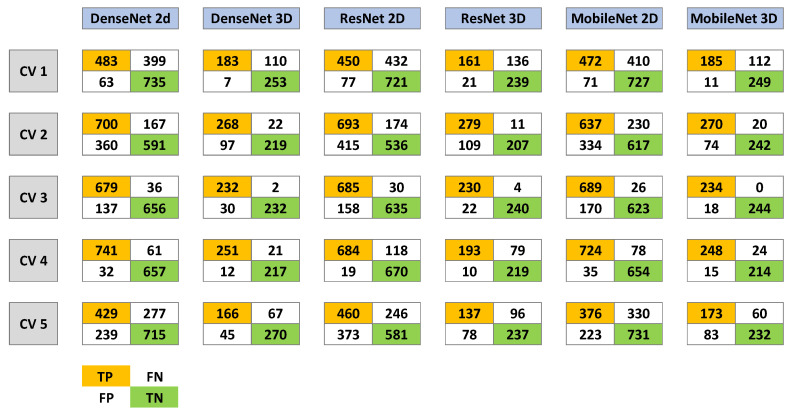
Confusion matrix values (TP = true positive, FN = false negative, FP = false positive, TN = true negative) across five cross-validation folds for all CNN models (2D and 3D).

After cross-validation, final models were retrained on the complete training set. All models exhibited a similar learning trajectory: a rapid decrease in binary cross-entropy loss and an increase in accuracy during the first 10 epochs, followed by convergence around epoch 15 after learning rate adjustments. In final performance metrics, 2D DenseNet and 2D ResNet achieved the lowest loss (~0.01), followed by 2D MobileNet and 3D ResNet (~0.03). Additionally, 3D DenseNet and 3D MobileNet converged at slightly higher loss values (~0.07). Accuracy and F1-scores reflected similar trends, with 2D models achieving marginally higher values overall compared with 3D counterparts during validation. Detailed results of the cross-validation process are shown in Figure 2 and Table 1. Paired fold-level comparison of 2D and 3D CNN cross-validation results, with a paired t-test and Cohen’s d, are shown in Table 2. Here, mean F1 scores and accuracy from the cross-validation runs of 2D and 3D versions of the same model are compared. Results are significantly better for the 3D architectures of the DenseNet and MobileNet models.

### 3.3. Unknown Data/Test Set Results

Independent evaluation was performed on a reserved test set comprising 10 scans (5 non-malignant and 5 malignant). Sensitivity, specificity, accuracy, and F1-score were calculated for each architecture (Table 3). The 3D DenseNet model achieved the highest overall diagnostic performance, with a specificity of 81%, sensitivity of 72%, and the highest F1-score (0.74). Other architectures demonstrated comparable classification results, with 3D variants consistently outperforming their 2D equivalents in both sensitivity and specificity.

## 4. Discussion

In this ex vivo study, we demonstrated the potential of OCT combined with AI-assisted image analysis to differentiate PDAC from non-malignant pancreatic tissue ex vivo. The results were consistent across multiple CNN architectures, with minimal performance variation between models. To our knowledge, this is the first study to apply deep learning to OCT imaging of pancreatic tissue and the first to directly compare 2D and 3D CNN approaches in this context.

Across all architectures, 3D models consistently outperformed their 2D counterparts, despite being trained on a smaller number of samples (C-scans instead of B-Scans). This finding highlights the superior capacity of volumetric OCT data to capture spatial and textural features relevant for tissue differentiation. Although 3D acquisition and analysis are more time-intensive, they yield more reliable diagnostic performance. Among the tested architectures, ResNet achieved the lowest overall performance but still produced acceptable results, with a specificity of 83% and sensitivity of 69% in its 3D configuration. For DenseNet121 and MobileNetV2, 3D models achieved significantly higher F1-score and accuracy compared to their 2D variants. In contrast, no statistically significant differences were detected between 2D and 3D configurations of ResNet50.

Our results are not directly comparable to previous studies on OCT-based pancreatic tissue characterization, as these did not employ AI-methods. For example, Van Manen et al. [13] examined 100 OCT images from specimens of 29 patients, with two pathologists scoring images as malignant or benign independently. They achieved a sensitivity of 72% and specificity of 74%, respectively, when compared to the corresponding H&E slides [13]. In an explorative study, Kist et al. [14] examined samples from 4 patients who underwent pancreatic surgery. They examined the feasibility of OCT for distinguishing benign, premalignant, and malignant pancreatic lesions, without conducting any formal statistical analysis. Additionally, Iftimia et al. [16] examined 66 fresh pancreatectomy specimens from patients with cystic lesions, which were split into training and test sets. Criteria developed using the first set were applied by three clinicians to classify OCT images in the test set and results were compared to histological examination, with sensitivity and specificity reaching values over 95%. Other studies focused on feasibility of OCT imaging in pancreatic tissues, such as a needle-based approach in hamster pancreata [15], in vivo scanning of canine pancreatobiliary systems [23] and other OCT-based approaches [24]. Further studies have been carried out on the pancreatobiliary system without specifically focusing on pancreas and related pathologies [17,18,25,26,27].

Nevertheless, our results are consistent with prior work applying AI-assisted OCT imaging in oncologic contexts. In previous studies, our group demonstrated high diagnostic accuracy using CNN-based analysis of OCT data, achieving F1-scores of 0.93 for differentiating colorectal liver metastases and healthy liver tissue [9] and 0.94 for intrahepatic cholangiocarcinoma [8]. Furthermore, Luo et al. achieved an area under the curve of 0.975 in distinguishing colorectal cancer from normal colon tissues, using OCT combined with ResNet in 43 tissue specimens [28]. Finally, Scholler et al. combined OCT with various AI-algorithms, including CNN, to reach accuracies of over 96% in distinguishing breast cancer from normal tissue in mastectomy specimens [29]. The lower accuracy observed in the current study can be partly attributed to the complex histoarchitecture of pancreatic tissue, which exhibits heterogeneous glandular structures, variable fat content, and collagen fibers, which can simulate PDAC. This complicates both OCT signal interpretation and CNN-based classification [13].

Although no prior work has compared 2D and 3D CNNs for pancreatic OCT data, similar studies in other tissues support our observations. For example, Rasel et al. compared 2D and 3D CNNs for glaucoma detection from retinal OCT volumes. They reported slightly superior performance for 2D models (AUC up to 0.96), likely due to overfitting in 3D models and limited dataset size [30]. Conversely, Tampu et al. evaluated thyroid OCT images and found that a 3D Vision Transformer outperformed 2D CNNs, achieving 90% accuracy and a Matthews correlation coefficient of 0.79 [31]. These findings collectively suggest that 3D architectures may provide diagnostic advantages when sufficient data and computational resources are available. These findings collectively suggest that 3D architectures may provide diagnostic advantages when sufficient data and computational resources are available. Direct comparisons between 2D and 3D CNN approaches for OCT data remain uncommon. A recent 2024 study investigating glaucoma detection from retinal OCT volumes reported that 2D CNN outperformed 3D models, likely reflecting dataset size and overfitting constraints rather than inherent architectural superiority [32]. To our knowledge, comparable 2D versus 3D analyses in OCT-based tissue characterization of pancreas have not yet been reported.

This study’s strengths include the use of histopathologically verified region-level labeling, the systematic evaluation of three CNN architectures across 2D and 3D input formats, and the implementation of repeated sampling and cross-validation for robust model assessment. Despite moderate classification performance, the findings establish a methodological foundation for future optimization and potential intraoperative application.

Several limitations must be acknowledged. First, the analyses were performed ex vivo; thus, the influence of physiological motion, perfusion, and temperature on in vivo imaging remains to be determined. Second, while 3D imaging provided superior accuracy, its acquisition and processing times may currently limit intraoperative feasibility. Third, all scans were acquired at a single center using the same OCT system under standardized ex vivo conditions. While this ensured methodological consistency, it limits technical variability and may restrict generalizability across different institutions, devices, operators, and clinical environments. Fourth, the relatively small cohort size constrained model generalizability. Although scan-level class distribution was balanced, the limited number of patients and acquisition settings may not fully capture the biological and technical heterogeneity of PDAC. Larger, multicenter datasets will therefore be essential for external validation. Finally, an important methodological limitation is that data splitting was performed at the scan level rather than at the patient level. As several scans originated from the same patient, overlap of patient data across training and test sets cannot be excluded. Future studies should ensure strict patient-level stratification to improve generalizability.

Overall, these findings support the feasibility of CNN-assisted OCT for rapid, label-free tissue differentiation in pancreatic surgery. A potential near-term clinical application could involve intraoperative assessment of resection margins to complement frozen section analysis, provided real-time acquisition and processing can be achieved. Additionally, integration into endoscopic ultrasound-guided platforms may allow enhanced characterization of suspicious pancreatic lesions during diagnostic procedures [10,17]. However, translation into clinical workflows requires several intermediate steps, including prospective in vivo validation, strict patient-level data separation, multicenter external validation, and benchmarking against established intraoperative diagnostic standards. Larger, prospective studies are necessary before routine clinical implementation can be considered.

## 5. Conclusions

This proof-of-concept study demonstrates that OCT combined with CNN-assisted image analysis enables reliable differentiation between PDAC and non-malignant pancreatic tissue ex vivo. The results highlight the potential of AI-assisted OCT for intraoperative application, offering a pathway to reduce operative time by minimizing reliance on frozen section diagnostics. While the results are promising, further validation in larger, prospectively designed in vivo studies is necessary before translation into intraoperative clinical workflows. Future research should focus on validating this approach in clinical settings, particularly for in vivo imaging and real-time assessment of resection margins.

## Figures and Tables

**Table 1:** Performance metrics for DenseNet121, ResNet50, and MobileNetV2 in 2D and 3D across five cross-validation (CV) folds and their respective mean values.

CNN, CV	Sensitivity/Recall	Specificity	PPV/Precision	NPV	Accuracy	F1-Score
DenseNet 2D CV1	0.55	0.92	0.88	0.65	0.73	0.68
DenseNet 2D CV2	0.81	0.62	0.66	0.78	0.71	0.73
DenseNet 2D CV3	0.95	0.83	0.83	0.95	0.89	0.89
DenseNet 2D CV4	0.92	0.95	0.96	0.92	0.94	0.94
DenseNet 2D CV5	0.61	0.75	0.64	0.72	0.69	0.62
**DenseNet 2D mean**	**0.77**	**0.81**	**0.80**	**0.80**	**0.79**	**0.77**
DenseNet 3D CV1	0.62	0.97	0.96	0.70	0.79	0.76
DenseNet 3D CV2	0.92	0.69	0.73	0.91	0.80	0.82
DenseNet 3D CV3	0.99	0.89	0.89	0.99	0.94	0.94
DenseNet 3D CV4	0.92	0.95	0.95	0.91	0.93	0.94
DenseNet 3D CV5	0.65	0.86	0.79	0.80	0.80	0.71
**DenseNet 3D mean**	**0.82**	**0.87**	**0.86**	**0.86**	**0.85**	**0.83**
ResNet 2D CV1	0.51	0.90	0.85	0.63	0.70	0.64
ResNet 2D CV2	0.80	0.56	0.63	0.75	0.68	0.70
ResNet 2D CV3	0.96	0.80	0.81	0.95	0.88	0.88
ResNet 2D CV4	0.85	0.97	0.97	0.85	0.91	0.91
ResNet 2D CV5	0.65	0.61	0.55	0.70	0.63	0.60
**ResNet 2D mean**	**0.75**	**0.77**	**0.76**	**0.78**	**0.76**	**0.75**
ResNet3D CV1	0.54	0.92	0.88	0.64	0.72	0.67
ResNet3D CV2	0.96	0.66	0.72	0.95	0.80	0.82
ResNet3D CV3	0.98	0.92	0.91	0.98	0.95	0.95
ResNet3D CV4	0.71	0.96	0.95	0.73	0.82	0.81
ResNet3D CV5	0.59	0.75	0.64	0.71	0.68	0.61
**ResNet 3D mean**	**0.76**	**0.84**	**0.82**	**0.80**	**0.79**	**0.77**
MobileNet 2D CV1	0.54	0.91	0.87	0.64	0.71	0.66
MobileNet 2D CV2	0.73	0.65	0.66	0.73	0.69	0.69
MobileNet 2D CV3	0.96	0.79	0.80	0.96	0.87	0.88
MobileNet 2D CV4	0.90	0.95	0.95	0.89	0.92	0.93
MobileNet 2D CV5	0.53	0.77	0.63	0.69	0.67	0.58
**MobileNet 2D mean**	**0.73**	**0.81**	**0.78**	**0.78**	**0.77**	**0.75**
MobileNet 3D CV1	0.62	0.96	0.94	0.69	0.78	0.75
MobileNet 3D CV2	0.93	0.77	0.78	0.92	0.84	0.85
MobileNet 3D CV3	1.00	0.93	0.93	1.00	0.96	0.96
MobileNet 3D CV4	0.91	0.93	0.94	0.90	0.92	0.93
MobileNet 3D CV5	0.74	0.74	0.68	0.79	0.74	0.71
**MobileNet 3D mean**	**0.84**	**0.87**	**0.86**	**0.86**	**0.85**	**0.84**

Reported metrics include sensitivity (recall), specificity, positive predictive value (PPV/precision), negative predictive value (NPV), accuracy, and F1-score. Mean values for each model across 5 CV-folds are shown in bold.

**Table 2:** Paired fold-level comparison of 2D and 3D CNN cross-validation results, with a paired t-test and Cohen’s d.

CNN	Metric	Mean Δ (3D vs. 2D)	95% CI of Δ	*p*-Value	Cohen’s d	CNN
DenseNet121	F1	0.061	[0.012–0.111]	0.026	1.547	DenseNet121
DenseNet121	Accuracy	0.040	[0.009–0.115]	0.032	1.442	DenseNet121
ResNet50	F1	0.028	[−0.072–0.128]	0.482	0.346	ResNet50
ResNet50	Accuracy	0.026	[−0.060–0.136]	0.344	0.480	ResNet50
MobileNetV2	F1	1.547	[0.018–0.168]	0.026	1.541	MobileNetV2
MobileNetV2	Accuracy	0.077	[0.007–0.147]	0.038	1.360	MobileNetV2

The difference in metrics between 3D and 2D models is given as Δ, with 95% confidence intervals.

**Table 3:** Classification performance of 2D and 3D CNN architectures on the test data.

CNN	Sensitivity	Specificity	Accuracy	F1-Score
Densenet121	0.71	0.68	0.69	0.68
Densenet121 3D	0.72	0.81	0.77	0.74
Mobilenet_v2	0.72	0.70	0.71	0.69
Mobilenet_v2 3D	0.60	0.86	0.74	0.67
ResNet50	0.69	0.70	0.70	0.68
ResNet50 3D	0.69	0.83	0.79	0.73

## Data Availability

Data used and generated during this study can be made available upon reasonable request to the corresponding author.

## Abbreviations

The following abbreviations are used in this manuscript:

2D	Two-dimensional
3D	Three-dimensional
AI	Artificial Intelligence
CNR	Contrast-to-Noise Ratio
CNN	Convolutional Neural Network
CV	Cross-Validation
DL	Deep Learning
H&E	Hematoxylin and Eosin
ML	Machine Learning
OCT	Optical Coherence Tomography
PDAC	Pancreatic ductal adenocarcinoma
